# Supplementary material for: Molecular and clinical characterization of a founder mutation causing G6PC3 deficiency
Source: medRxiv. 2024 May 14:2024.05.13.24307299. Preprint. [Version 1] doi: 10.1101/2024.05.13.24307299 (PMC11118594; doi:10.1101/2024.05.13.24307299)
Supplement: 1 [file NIHPP2024.05.13.24307299V1-supplement-1.pdf]

medRxiv preprint doi: https://doi.org/10.1101/2024.05.13.24307299; this version posted May 14, 2024. The copyright holder for this preprint (which was not certified by peer review) is the author/funder, who has granted medRxiv a license to display the preprint in perpetuity.
It is made available under a CC-BY 4.0 International license .

Supplementary material

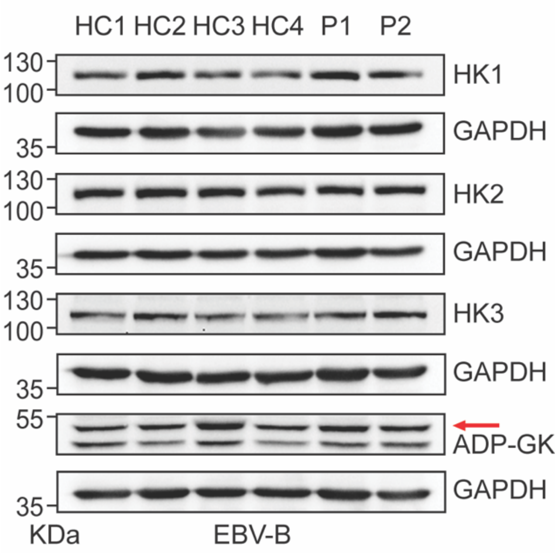

Supplementary Figure 1: Hexokinase expression in EBV-B cells. Western blot expression of the four isoforms of hexokinase in EBV-B cells. GAPDH was used as the loading control.
